# Bioprótese Valvar Porcina: Um Legado de Mario Vrandecic

**DOI:** 10.36660/abc.20201110

**Published:** 2021-04-08

**Authors:** Erika Correa Vrandecic, Ektor Correa Vrandecic, Bayard Gontijo, Rossana Dall’Orto Elias, Braulio Roberto Gonçalves Marinho Couto, Marcus Vinicius Bolivar Malachias

**Affiliations:** 1 Biocor Instituto Nova LimaMG Brasil Biocor Instituto, Nova Lima, MG - Brasil; 2 Faculdade de Ciências Médicas de Minas Gerais Belo HorizonteMG Brasil Faculdade de Ciências Médicas de Minas Gerais, Belo Horizonte, MG - Brasil

**Keywords:** Doença das Valvas Cardíacas/cirurgia, Implante de Prótese de Valva Cardíaca, Biopróteses/tendências, Mario Vrandecic

A doença valvar cardíaca ocupa atualmente os holofotes da medicina cardiovascular em face dos recentes avanços das técnicas de imagens e emergentes possibilidades terapêuticas, atraindo a atenção de médicos, pesquisadores, fabricantes de dispositivos e investidores.^1^ O Brasil ocupa um lugar de destaque internacional na história e no desenvolvimento tecnológico de substitutos valvares utilizados no tratamento dessa enfermidade.

O primeiro implante mundial de uma bioprótese valvar suína, comercialmente disponível, aconteceu em outubro de 1968.^2^ Cerca de meio século depois, em setembro de 2019, faleceu o médico e cientista Mario Vrandecic, criador da única bioprótese cardíaca de tecido porcino produzida no Brasil e aprovada na agência norte-americana Food and Drug Adminstration (FDA), há décadas globalmente utilizada no tratamento da doença cardíaca valvar.

Nesse artigo, destacamos a história do seu criador e as evidências de efetividade e segurança da bioprótese valvar Biocor, hoje denominada *St. Jude Medical Biocor* (St. Jude Medical, Inc., St Paul, MN).^3^^–^^16^

Mario Vrandecic, de ascendência croata e nascido na Bolívia, cursou Medicina na Faculdade de Medicina da Universidade Federal de Minas Gerais (FM/UFMG). Especializou-se em cirurgia geral e cardiovascular nos Estados Unidos da América (EUA), onde serviu ao exército americano como cirurgião, inclusive durante a guerra do Vietnã. Retornou definitivamente ao Brasil em 1976 e passou a atuar como professor da FM/UFMG e cirurgião cardiovascular na Santa Casa de Belo Horizonte, entre outros hospitais.

Tendo realizado pesquisas com tecidos biológicos durante a sua residência nos EUA, em 1981 criou a Biocor Indústria, empresa na qual desenvolveu uma bioprótese valvar cardíaca de tecido porcino, entre outras patentes. Inicialmente usada no Brasil, na América Central e na Ásia, a bioprótese logo obteve o *CE Marking*, passando a ser utilizada na Europa e, posteriormente, com a aprovação pelo FDA, também nos EUA. Recebeu homenagens de várias sociedades científicas e de entidades da área de inovação nacionais e internacionais. Em 1997, a Biocor Indústria foi vendida à empresa norte-americana St. Jude Medical, sendo posteriormente incorporada no ano de 2016 à Abbott Laboratories.

Após quase 40 anos de uso clínico, as evidências de seguimentos de curto, médio e longo prazos demonstram efetividade, durabilidade e segurança da referida bioprótese valvar em séries nacionais e de outros países (Tabela 1).^3^^–^^16^ Em um dos seguimentos mais longos, Mykén e Bech-Hansen, avaliaram 1.712 pacientes que receberam a bioprótese Biocor porcina no Sahlgrenska University Hospital, em Gotemburgo, na Suécia, demonstrando uma sobrevida livre de mortalidade devido à falência valvar após 20 anos de 84,3% ± 6,9% e 88,0% ± 4,0%, para implantes em posições aórtica e mitral, respectivamente^16^ (Tabela 1).

**Tabela 1:** Desfechos clínicos observados em pacientes que receberam bioprótese porcina Biocor em posições aórtica e mitral*

Autor/referência	Período de acompanhamento	Posição da bioprótese	Tamanho da amostra (n)	Desfecho	Resultado observado
Vrandecic^3^^,^^4^	Março 1981– março 1988(48 [1 a 84] meses)	Aórtica + Mitral	1.713	Mortalidade hospitalar	6,1%
				Sobrevida após 7 anos	97,1%
		Aórtica	385	Complicações tardias	13,2%
				Sobrevida livre de falência valvar após 7 anos	96,9%
		Mitral	716	Complicações tardias	14,2%
				Sobrevida após 7 anos	95,2%
Gontijo-Filho^5^	Maio 1990 – março 1992 (9 [1 a 22] meses)	Aórtica	81	Mortalidade hospitalar	4,9%
Gontijo-Filho^6^	Junho 1990 – janeiro 1993	Aórtica *stentless* em alterações do anel aórtico	16	Mortalidade hospitalar	6,3%
Vrandecic^7^	Março 1992 – março 1993 (6 [1 a 12] meses)	Mitral	38	Mortalidade hospitalar	0%
				Retroca valvar	3,8%
Vrandecic^8^	Maio 1990 –dezembro 1993	Áortica *stentless*	120	Mortalidade hospitalar	5%
				Retroca valvar	4%
Vrandecic^9^	(14 [1 a 26] meses)	Mitral *stentless*	85	Mortalidade hospitalar	0%
				Retroca valvar	6%
Vrandecic^10^	Março 1992 –dezembro 1995 (26 [3 a 45] meses)	Mitral *stentless*	108	Mortalidade hospitalar	6,5%
				Retroca valvar	12,5%
Vrandecic^11^	Março 1992 – agosto 1996 (29 [2 a 54] meses)	Mitral *stentless*	120	Mortalidade hospitalar	6,5%
				Retroca valvar	14,3%
Vrandecic^12^	Janeiro 1990 – junho 1999 (54 [3 a 114] meses)	Aórtica *stentless* vs *stented*	407	Sobrevida em 8 anos	71,8% ± 0,7% (*stentless*) vs 62,9% ± 13,4% (*stented*)
Kirali^13^	Janeiro 1985 – junho 1999 (10 [1 a 15] anos)	Mitral	158	Mortalidade em 30 dias	4,4%
				Retroca valvar	14%
				Sobrevida livre em 5 anos	83,7% ± 3%
				Sobrevida em 13 anos	77,8% ± 3,4%
				Sobrevida livre de falência valvar após 5 anos	95,5% ± 1.8%
				Sobrevida livre de falência valvar após 13 anos	64,8% ± 5,3%
				Sobrevida livre de reoperação, devido falência valvar após 5 anos	98,4% ± 1,1%
				Sobrevida livre de reoperação, devido falência valvar após 10 anos	89,2% ± 2,9%
				Sobrevida livre de reoperação, devido falência valvar após 14 anos	76,8% ± 7,9%
Pomerantzeff^14^	Março 1983 – dezembro 2000	Mitral	546	Mortalidade hospitalar	9,5%
				Sobrevida em 15 anos	45% ± 15,8%
				Sobrevida livre de reoperação, devido falência valvar após 15 anos	33,9% ± 10,4%
Eichinger^15^	Janeiro 1985 –dezembro 2006 (8 [1 a 21] anos)	Aórtica	455	Mortalidade em 30 dias	5.3%
				Sobrevida em 5 anos	74,7% ± 2,0%
				Sobrevida em 10 anos	44,9% ± 2,4%,
				Sobrevida em 15 anos	20,9% ± 2,5%
				Sobrevida em 20 anos	9,4% ± 2,8%.
				Sobrevida livre de falência valvar após 5 anos	97,5% ± 0,8%
				Sobrevida livre de falência valvar após 10 anos	93,1% ± 1,7%
				Sobrevida livre de falência valvar após 15 anos	88,4% ± 3,5%
				Sobrevida livre de falência valvar após 20 anos	70,3% ± 10,9%
				Sobrevida livre de reoperação, devido falência valvar após 5 anos	95,9% ± 1%
				Sobrevida livre de reoperação, devido falência valvar após 10 anos	91,9% ± 1,6%
				Sobrevida livre de reoperação, devido falência valvar após 15 anos	90,6% ± 2,1%
				Sobrevida livre de reoperação, devido falência valvar após 20 anos	86,5% ± 4,5%
Mykén^16^	Janeiro 1983 – janeiro 2003 [média 6 anos]	Aórtica	1.518	Mortalidade hospitalar	5,1%
				Incidência de reoperação	0,9%/paciente-ano
				Sobrevida livre de mortalidade, devido falência valvar após 20 anos	84,3% ± 6.9%
				Sobrevida livre de reoperação, devido falência valvar após 20 anos	61,1% ± 8,5%
		Mitral	194	Mortalidade hospitalar	12,9%
				Incidência de reoperação	0,9%/paciente-ano
				Sobrevida livre de mortalidade, devido falência valvar após 20 anos	88,0% ± 4,0%
				Sobrevida livre de reoperação, devido falência valvar após 20 anos	79,3% ± 6,0%

* Dados de 14 publicações que avaliaram os desfechos de curto, médio e longo prazos das biopróteses porcinas Biocor, publicados entre 1988 e 2008.^3^^–^^16^

Mario Vrandecic fundou ainda, em 1985, o Biocor Instituto, localizado em Nova Lima, região metropolitana de Belo Horizonte (MG). Inicialmente dedicado às doenças cardiovasculares, o hospital logo evoluiu para ser um importante centro de Medicina de alta complexidade. O hospital tem sido o responsável pela especialização e atuação de muitas gerações de cardiologistas, cirurgiões cardíacos, médicos de diversas especialidades e outros profissionais de saúde, além de ser referência em assistência de qualidade à população do estado, com importantes certificações nacionais e internacionais. A gestão de Mario Vrandecic foi pautada na ética, na geração de confiança, na qualificação de pessoas e na educação continuada. O seu legado simboliza um exemplo de humanismo e dedicação à Medicina, um marco de inovação em ciência cardiovascular e uma prova da potencialidade biotecnológica do país.
