# Supplementary material for: Bioprótese Valvar Porcina: Um Legado de Mario Vrandecic
Source: Arq Bras Cardiol. 2021 Apr 8;116(4):840–3. [Article in Portuguese] doi: 10.36660/abc.20201110 (PMC8121389; doi:10.36660/abc.20201110)
Supplement: Translation

# Translation: Porcine Valve Bioprosthesis: a Legacy from Mario Vrandecic

**Authors:** Erika Correa Vrandecic, Ektor Correa Vrandecic, Bayard Gontijo, Rossana Dall’Orto Elias, Braulio Roberto Gonçalves Marinho Couto, Marcus Vinicius Bolivar Malachias

**Affiliations:** 1 Nova LimaMG Brazil Biocor Institute, Nova Lima, MG - Brazil; 2 Belo HorizonteMG Brazil Faculdade de Ciências Médicas de Minas Gerais, Belo Horizonte, MG - Brazil

**Keywords:** Heart Valve Diseases/surgery, Heart Valve Prosthesis Implantation, Bioprosthesis/trends, Mario Vranderic

Heart valve disease currently occupies the spotlight in cardiovascular medicine due to recent advances in imaging techniques and emerging therapeutic possibilities, attracting the attention of physicians, researchers, device manufacturers, and investors.^1^ Brazil has a prominent international role in the history and technological development of prosthetic heart valves used to treat this disease.

The first worldwide implantation of a commercially available porcine valve bioprosthesis occurred in October 1968.^2^ About half a century later, in September 2019, passes on the physician and scientist Mario Vrandecic, creator of the only heart valve bioprosthesis made from porcine tissue produced in Brazil and approved by the United States (US) Food and Drug Administration (FDA), globally used for decades in the treatment of heart valve disease.

In this article, we highlight the history of the creator and the evidence of effectiveness and safety of the Biocor valve bioprosthesis, known today as St. Jude Medical Biocor (St. Jude Medical, Inc., St Paul, MN).^3^^–^^16^

Mario Vrandecic, of Croatian ancestry and a native of Bolivia, studied medicine at the School of Medicine at *Universidade Federal de Minas Gerais* (FM/UFMG). He specialized in general and cardiovascular surgery in the US, where he served in the US army as a surgeon, including during the Vietnam War. He returned permanently to Brazil in 1976 and started working as a professor at FM/UFMG and as a cardiovascular surgeon at *Santa Casa de Belo Horizonte*, among other hospitals.

Having conducted research on biological tissues during his residency in the US, Vrandecic created in 1981 the Biocor Indústria, where he developed a heart valve bioprosthesis made of porcine tissue, among other patents. Initially used in Brazil, Central America, and Asia, the bioprosthesis soon obtained CE Marking, and was used in Europe and, with later FDA approval, also in the US. He received honors from several scientific societies and national and international entities in the area of innovation. In 1997, Biocor Indústria was sold to the US company St. Jude Medical, which was acquired by Abbott Laboratories later in 2016.

After almost 40 years of clinical use, evidence of short-, medium-, and long-term follow-up has demonstrated the effectiveness, durability, and safety of the valve bioprosthesis in national and international series (Table 1).^3^^–^^16^ In one of the most prolonged follow-up periods, Mykén and Bech-Hansen evaluated 1712 patients who received the Biocor porcine bioprosthesis at Sahlgrenska University Hospital (Gothenburg, Sweden), demonstrating rates of freedom from valve-related death at 20 years of 84.3 ± 6.9% and 88.0% ± 4.0% for aortic and mitral valve replacement, respectively^16^ (Table 1).

**Table 1:** Clinical outcomes observed in patients who received aortic and mitral valve replacement with the Biocor porcine bioprosthesis*

Author/Reference	Follow-up period	Bioprosthesis position	Sample size (n)	Outcome	Observed outcome
Vrandecic ^3^^.^^4^	March 1981 - March 1988 (48 [1 to 84] months)	Aortic + Mitral	1713	In-hospital mortality	6.1%
				Survival at 7 years	97.1%
		Aortic	385	Late complications	13.2%
				Freedom from valve dysfunction at 7 years	96.9%
		Mitral	716	Late complications	14.2%
				Survival at 7 years	95.2%
Gontijo-Filho^5^	May 1990 - March 1992 (9 [1 to 22] months)	Aortic	81	In-hospital mortality	4.9%
Gontijo-Filho^6^	June 1990 -January 1993	Aortic "stentless" in aortic annulus abnormalities	16	In-hospital mortality	6.3%
Vrandecic^7^	March 1992 -March 1993 (6 [1 to 12] months)	Mitral	38	In-hospital mortality	0%
				Valve reoperation	3.8%
Vrandecic^8^	May 1990 -December 1993	Aortic "stentless"	120	In-hospital mortality	5%
				Valve reoperation	4%
Vrandecic^9^	(14 [1 to 26] months)	Mitral “stentless”	85	In-hospital mortality	0%
				Valve reoperation	6%
Vrandecic^10^	March 1992 -December 1995 (26 [3 to 45] months)	Mitral "stentless"	108	In-hospital mortality	6.5%
				Valve reoperation	12.5%
Vrandecic^11^	March 1992 -August 1996 (29 [2 to 54] months)	Mitral “stentless"	120	In-hospital mortality	6.5%
				Valve reoperation	14.3%
Vrandecic^12^	January 1990 -June 1999 (54 [3 to 114] months)	Aortic "stentless" vs. "stented"	407	8-year survival	71.8 ± 0.7% (“stentless”) vs. 62.9 ± 13.4% (“stented”)
Kirali^13^	January 1985 -June 1999 (10 [1 to 15] years)	Mitral	158	30-day mortality	4.4%
				Valve reoperation	14%
				5-year cumulative survival	83.7±3%
				13-year cumulative survival	77.8±3.4%
				Freedom from structural valve deterioration at 5 years	95.5±1.8%
				Freedom from structural valve deterioration at 13 years	64.8±5.3%
				Freedom from structural valve deterioration-related reoperation at 5 years	98.4±1.1%
				Freedom from structural valve deterioration-related reoperation at 10 years	89.2±2.9%
				Freedom from structural valve deterioration-related reoperation at 14 years	76.8±7.9%
Pomerantzeff^14^	March 1983 - December 2000	Mitral	546	In-hospital mortality	9.5%
				15-year survival	45±15.8%
				Freedom from structural valve deterioration-related reoperation at 15 years	33.9 ± 10.4%
Eichinger^15^	January 1985 -December 2006 (8 [1 to 21] years)	Aortic	455	30-day mortality	5.3%
				5-year survival	74.7% ± 2.0%
				10-year survival	44.9% ± 2.4%,
				15-year survival	20.9% ± 2.5%
				20-year survival	9.4% ± 2.8%.
				Freedom from nonstructural valve dysfunction at 5 years	97.5 % ± 0.8%
				Freedom from nonstructural valve dysfunction at 10 years	93.1% ± 1.7%
				Freedom from nonstructural valve dysfunction at 15 years	88.4% ± 3.5%
				Freedom from nonstructural valve dysfunction at 20 years	70.3% ± 10.9%
				Freedom from reoperation due to structural valve deterioration at 5 years	95.9% ± 1%
				Freedom from reoperation due to structural valve deterioration at 10 years	91.9% ± 1.6%
				Freedom from reoperation due to structural valve deterioration at 15 years	90.6% ± 2.1%
				Freedom from reoperation due to structural valve deterioration at 20 years	86.5% ± 4.5%
Mykén^16^	January 1983 -January 2003 (mean 6 years)	Aortic	1518	In-hospital mortality	5.1%
				Incidence of reoperation	0.9%/patient-year
				Freedom from valve-related death at 20 years	84.3 ± 6.9%
				Freedom from reoperation due to structural valve deterioration at 20 years	61.1% ± 8.5%
		Mitral	194	In-hospital mortality	12.9%
				Incidence of reoperation	0.9%/patient-year
				Freedom from valve-related death at 20 years	88.0% ± 4.0%
				Freedom from reoperation due to structural valve deterioration at 20 years	79.3% ± 6.0%

* Data from 14 publications evaluating short-, medium-, and long-term outcomes with the Biocor Porcine Bioprosthesis, published between 1988 and 2008.3-16

Mario Vrandecic also founded, in 1985, the Biocor Institute, located in Nova Lima, metropolitan region of Belo Horizonte, MG. Initially dedicated to cardiovascular diseases, the hospital soon evolved into an important high-complexity medical center. The hospital has been responsible for specialization and work of many generations of cardiologists, cardiac surgeons, physicians of various specialties, and other health care professionals, in addition to being a reference in quality assistance to the population of the state, with important national and international certifications. Mario Vrandecic's management was based on ethics, generation of trust, qualification of individuals, and continuing education. His legacy symbolizes an example of humanism and dedication to medicine, a landmark of innovation in cardiovascular science, and a testament to the country's biotechnological potential.
